# Gγ1, a Downstream Target for the *hmgcr*-Isoprenoid Biosynthetic Pathway, Is Required for Releasing the Hedgehog Ligand and Directing Germ Cell Migration

**DOI:** 10.1371/journal.pgen.1000333

**Published:** 2009-01-09

**Authors:** Girish Deshpande, Anuradha Godishala, Paul Schedl

**Affiliations:** Department of Molecular Biology, Princeton University, Princeton, New Jersey, United States of America; University of California San Francisco, United States of America

## Abstract

The isoprenoid biosynthetic pathway leading from the production of mevalonate by HMGCoA reductase (Hmgcr) to the geranylation of the G protein subunit, Gγ1, plays an important role in cardiac development in the fly. Hmgcr has also been implicated in the release of the signaling molecule Hedgehog (Hh) from *hh* expressing cells and in the production of an attractant that directs primordial germ cells to migrate to the somatic gonadal precursor cells (SGPs). The studies reported here indicate that this same *hmgcr*→Gγ1 pathway provides a novel post-translational mechanism for modulating the range and activity of the Hh signal produced by *hh* expressing cells. We show that, like *hmgcr*, *gγ1* and *quemao* (which encodes the enzyme, geranylgeranyl diphosphate synthetase, that produces the substrate for geranylation of Gγ1) are components of the *hh* signaling pathway and are required for the efficient release of the Hh ligand from *hh* expressing cells. We also show that the *hmgcr*→Gγ1 pathway is linked to production of the germ cell attractant by the SGPs through its ability to enhance the potency of the Hh signal. We show that germ cell migration is disrupted by the loss or gain of *gγ1* activity, by *trans*-heterozygous combinations between *gγ1* and either *hmgcr* or *hh* mutations, and by ectopic expression of dominant negative Gγ1 proteins that cannot be geranylated.

## Author Summary

Previous studies have shown that HMGCoA reductase (Hmgcr) is required for the production of a germ cell attractant by the somatic gonadal precursor cells (SGPs) and for the release of the Hedgehog (Hh) ligand by *hh* expressing cells. However, it was not clear what role mevalonate, the biosynthetic product of Hmgcr, played in either of these processes or whether the *hmgcr*-dependent germ cell attractant corresponds to the Hh ligand (which is known to be expressed by the SGPs). We show here that the downstream target for Hmgcr both in generating the germ cell attractant and in releasing the Hh ligand is the G protein, Gγ1. Gγ1 must be geranylated in order to function, and the substrate for this posttranslational modification, geranylgeranyl-pyrophosphate, is one of the biosynthetic products of mevalonate. In addition to demonstrating a critical role for Gγ1 (as well as the *hmgcr* isoprenoid biosynthetic pathway) in releasing Hh from *hh* expressing cells, our findings provide additional evidence that Hh protein produced by the SGPs is an *hmgcr*-dependent germ cell attractant.

## Introduction

Two distinct cell types, the primordial germ cells and the somatic gonadal precursor cells (SGPs), coalesce to form the *Drosophila* embryonic gonad (for review, see [1],[2]). These cells arise in different regions of the embryo and are specified by completely different mechanisms. The SGPs are derived from the lateral mesoderm in parasegments 10–13 during mid-embryogenesis and are specified by the input from a combination of cell-cell signaling pathways and zygotic patterning genes [3],[4]. By contrast, the primordial germ cells, or pole cells, are formed on the outside surface of the embryo at the posterior end during the syncitial blastoderm stage and are specified by determinants localized in the posterior pole plasm during oogenesis [5],[6]. In order for pole cells to assemble into a gonad with the SGPs, they must traverse from the posterior end into the middle of the embryo and then subsequently move to the lateral mesodermal cell layer, which contains the newly formed SGPs. This is a multistep process that begins at gastrulation when the pole cells are carried into the interior of the embryo by the midgut invagination [1],[2]. They then pass through the midgut epithelium, and move along the surface of the midgut until they split into two groups. The germ cells in each group migrate laterally and this brings them into contact with the gonadal mesoderm on either side of the embryo. The germ cells align themselves in a row with the SGPs in parasegments 10-13 and these juxtaposed cells coalesce into the embryonic gonad.

Analysis of the different migration steps has suggested that a combination of repulsive and attractive cues guide germ cell migration through the midgut and toward the somatic gonadal mesoderm. Repulsive clues, whose production depends upon Wunen and Wunen2, are thought to hasten the movement of the germ cells away from the midgut epithelium [7],[8]. Once the germ cells exit the midgut and migrate along its surface, attractive cues produced by the SGPs are thought to entice the germ cells towards that lateral mesoderm and promote their subsequent association with the SGPs.

One of the first genes implicated in the production of the germ cell attractant by the SGPs was *hmgcr* [9]. *hmgcr* is initially expressed broadly in the embryonic mesoderm; however, by the time germ cells commence their migration into the mesoderm, *hmgcr* expression is largely restricted to the SGPs [9]. In *hmgcr* mutants germ cells fail to migrate towards the SGPs and instead either remain associated with the midgut or scatter through the mesoderm. Conversely, ectoptically expressed *hmgcr* can induce germ cells to migrate towards tissues expressing the Hmgcr protein. Another gene that functions to induce migration towards the SGPs encodes the signaling molecule *hedgehog* (*hh*) [10]. Both ectopic expression of Hh and mutations that compromise the production or transmission of the Hh ligand by the SGPs induce mismigration. Since Hh functions as a morphogen in other contexts, one explanation for its effects on germ cell migration is that it acts indirectly by inducing cells to assume a SGP identity so that they can produce the actual attractant. However, a number of findings argue that Hh acts directly as an attractant. For one, the two known receptors of the Hh signal, Patched (Ptc) and Smoothened (Smo) are required in the germ cells for their proper migration. In the absence of the Hh ligand, the transmembrane receptor Ptc inhibits the 7-pass transmembrane protein Smo from mediating signal transduction [11]–[13]. When Hh binds to the Ptc receptor, the physical association between these two proteins is thought to relieve the negative influence of Ptc resulting in the relocalization of Smo to the cell membranes, and this in turn activates the signal transduction cascade downstream of the Hh signal. Consistent with their reciprocal functions in *hh* signaling, germ cells compromised for *ptc* or *smo* activity behave differently. For *ptc* the germ cells clump prematurely near the midgut as if they had already received the full Hh signal. For *smo* the germ cells behave as if they are ‘signal-blind’ and scatter randomly in the posterior of the embryo [10]. A second line of evidence supporting a direct role for Hh as the germ cell attractant comes from the discovery that *hgmcr* is required for the release of the Hh ligand by *hh* expressing cells. In embryos compromised for *hmgcr* activity [14], Hh is inappropriately retained in the *hh* expressing cells. Conversely, the range and strength of the Hh signal can be substantially enhanced by ectopic expression of *hmgcr*. Critically, ectopic *hmgcr* only had an effect on *hh* signaling when it was expressed in cells that normally produce the Hh ligand, while there was no effect when *hmgcr* is ectopically expressed in cells that normally receive the Hh ligand.

One important issue left unresolved by these studies is how *hmgcr* promotes the release of the Hh ligand by *hh* expressing cells. The *hmgcr* gene encodes HMGCoA reductase which is responsible for the conversion of 3-hydroxy-3-methylglutaryl coenzyme A to mevalonate. In mammals, mevalonate is a precursor for cholesterol which is used in the modification of the Hh protein. However, providing precursors for cholesterol biosynthesis is not a likely function for *hmgcr* in the *hh* signaling pathway as the genes encoding the enzymes required to synthesize cholesterol from appear to be absent in flies [15]. Mevalonate is also a precursor for many different compounds including carotenoids, isoprenoids, ubiquitones and vitamins A and E [16]. Recent studies by Santos and Lehmann [15] on the role of *hmgcr* in germ cell migration have implicated the isoprenoid branch of the mevalonate precursor pathway. The isoprenoids farnsyl-pyrophosphate (FPP) and geranylgeranyl-pyrophosphate (GGPP) are used in the posttranslational modification of proteins and are covalently attached to the C terminus of target proteins by farnysyl transferase and type I or type II geranylgeranyl transferases respectively. Santos and Lehmann showed that mutations in farnesyl-diphosphate synthetase (*fpps*) (which synthesizes FPP), geranylgeranyl diphosphate synthetase (*qm*) (which in turn converts FPP to GGPP), and geranylgeranyl transferase type I (*β-ggt1*) disrupt germ cell migration. They also found that germ cell migration is perturbed when *fpps* and *qm* are ectopically expressed. Though the effects were much less dramatic than observed for ectopic *hmgcr*, this is not altogether unexpected since these two genes differ from *hmgcr* in that they are widely expressed in mid-to-late embryogenesis.

While these findings indicate that the pathway leading from *hmgcr* to GGPP is important in germ cell migration because some critical target protein requires geranylation, the identity of this protein and the nature of its function in the production of the germ cell attractant remain to be established. Additionally, Santos and Lehmann [15] did not test whether *hh* signaling also depends upon this same isoprenoid biosynthetic pathway. Thus, the possibility remains open, especially if there is another germ cell attractant besides Hh, that *hmgcr* has some other function in *hh* signaling beside the production of isoprenoids. A possible answer to these questions comes from recent studies on cardiac development in flies. Hmgcr and downstream enzymes in the mevalonate pathway are required in cardioblasts to ensure their proper adhesion to the neighboring pericardial cells. Yi et al. [17] found that the endpoint for the isoprenoid branch of the *hmgcr* mevalonate pathway in heart development is the geranylgeranylation of the heterotrimeric G protein γ subunit 1 (Gγ1) [17],[18]. The C-terminus of the *Drosophila* Gγ1 protein has the isoprenylation CAAX motif sequence, Cys-Thr-Val-Leu. The leucine residue at the terminal position (X) specifies lipid modification by geranylgeranylation. Gγ1 requires this modification for membrane association and is inactive when geranylation is blocked. Significantly, *gγ1* would be a quite plausible downstream target for *hmgcr* activity in the *hh* signaling pathway (and thus in the production of the germ cell attractant). Though heterotrimeric G proteins are normally thought to mediate the transduction of extracellular signals by G-protein coupled receptors, recent studies indicate that these G protein complexes have other intercellular functions. In particular, the Gγ1:Gβ heterodimer together with Gα have been implicated in the transport of cargo from the *trans-*Golgi network (TGN) to the basolateral plasma membrane [19]–[21]. The involvement of machinery targeting proteins to the basolateral membrane from the TGN would make sense in the context of *hh* signaling as autoprocessed and fully modified Hh protein is found to preferentially accumulate in a punctate pattern along the basolateral membranes of Hh expressing cells in the embryonic ectoderm [22]–[24]. This protein is then released from the cell, through a Dispatched (Disp) dependent mechanism that is thought to involve translocation of the Hh puncta from their docking sites along the basolateral membranes to the apical membrane [25],[26]. In the studies reported here we have asked whether the *hmgcr→*Gγ1 pathway is important for *hh* signaling and whether *gγ1* is required for proper germ cell migration as is the case for *hmgcr*.

## Results

### *gγ1* Mutations Suppress the Gain-of-Function Wing Phenotypes of *hh^Mrt^*

To test whether *gγ1* is a component of the *hh* signaling pathway, we took advantage of the *hh^Moonrat^* (*hh^Mrt^*) mutation [27]. *hh^Mrt^* is a dominant gain-of-function *hh* allele that disrupts patterning of the wing as a heterozygote and is lethal as a homozygote. In wild type wing discs, *hh* expression is confined to the posterior compartment and it orchestrates wing development by signaling to cells in the anterior compartment along the compartment boundary to upregulate the expression target genes such as *ptc* and *decapentaplegic* (*dpp*). In *hh^Mrt^/+* animals, in addition to being expressed normally in the posterior compartment, *hh* is ectopically activated in the anterior compartment of the wing disc. As a result *dpp* is expressed in a pattern that leads to overgrowth of the anterior tissues and the partial duplication of distal wing structures. The anterior-to-posterior transformations induced by the *hh^Mrt^* allele can be dominantly suppressed by mutations in *hh* signaling pathway genes like *disp* and *hmgcr* that are required to promote *hh* signaling in the sending cells. The gain-of-function wing phenotype can also be suppressed by mutations in genes like *toutvelu* (*ttv*) that are required to promote *hh* signaling in the receiving cell (unpublished data).

**Figure 1:**
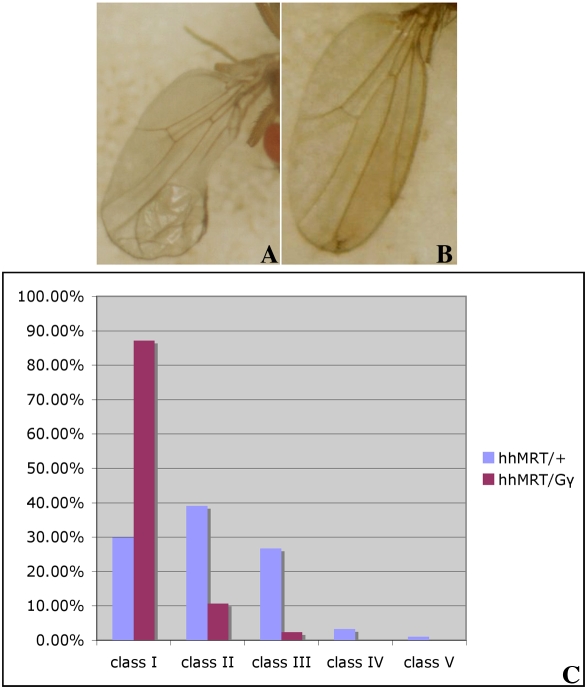
*gγ1* can dominantly suppress the wing abnormalities including pattern duplication induced by *hh ^MRT^*. Panel A shows an example of Class III type of wing defects induced by ectopic expression of Hh in the anterior compartment in *hh ^MRT^* animals. Panel B shows a wing from an animal of the genotype *hh ^MRT^*/*gγ1*. Almost complete suppression (Classified as Class I or II) of the wing phenotype can be seen. Panel C shows a graphic representation of the suppression of the wing defects. Roughly 250 single wing blades of the indicated genotypes were analyzed and classified into 5 different categories depending on the severity of the phenotype as previously described in Felsenfeld and Kennison, [27]. All the experimental as well as control crosses testing *hh^MRT^* suppression were carried out at 18°C.

If *gγ1* functions as the downstream target for *hmgcr* in the *hh* signaling pathway, then mutations in *gγ1* would be expected to dominantly suppress the *hh^Mrt^* wing defects. To test for suppression we used two different *gγ1* mutants. The first, *gγ1^N159^*, is an EMS induced mutation [28]. The *gγ1* open reading frame encodes a protein of 70 amino acids and this mutation inserts a stop codon at amino acid 59. The second, *gγ1^k0817^*, has a P-element insertion in the splice donor of the first *gγ1* exon and produces aberrant transcripts. To assess the effects of these *gγ1* mutations, the *Mrt* wing blades were assigned to 5 different classes based on the severity of the wing defects, with I being wild type (not shown), and V being the most severely deformed wing (not shown, for details see 27). Under the conditions of this experiment about 70**%** of the *hh^Mrt^/+* flies were abnormal (see the class III wing in panel A of Figure 1). By contrast when the *hh^Mrt^/+* flies were heterozygous for *gγ1^N159^* quite strong suppression was observed and more than 80**%** of the wings belonged to class I (panel B)**.** The suppression of the *Mrt* gain-of-function phenotype does not appear to be due to some non-specific background effect as the wing defects could also be dominantly suppressed by *gγ1^k0817^* (data not shown). Thus like *hmgcr* and other factors that function to promote *hh* signaling, *gγ1* shows genetic interactions with the *hh^Mrt^*. Moreover, the extent of suppression is similar to that observed previously with *hmgcr* [14].

### *gγ1* Is Required To Maintain *wg* and *en* Expression

The dominant suppression of the *Mrt* wing phenotypes suggests that like *hmgcr*, *gγ1* functions in *hh* signaling. To test this possibility further we examined *wingless* (*wg*) expression during embryogenesis. In wild type embryos, *wg* stripes are activated by the pair-rule genes at the onset of gastrulation. Once the pair-rule gene products decay later in embryogenesis the maintenance of the *wg* expression depends upon *hh* signaling by the cells immediately posterior to the *wg* stripe and in *hh* mutants *wg* expression begins disappearing by stage 10/11 of embryogenesis. Maintenance of the *wg* stripes also requires *hmgcr* activity and in *hmgcr* mutant embryos the stripes begin to fade around stage 11. However, unlike *hh* mutants, residual *wg* expression can still be detected in older *hmgcr* mutant embryos. Since maternal and zygotic *hmgcr* activity cannot be completely eliminated, this difference likely reflects (at least in part) the presence of residual Hmgcr in the *hmgcr* mutant embryos.

**Figure 2:**
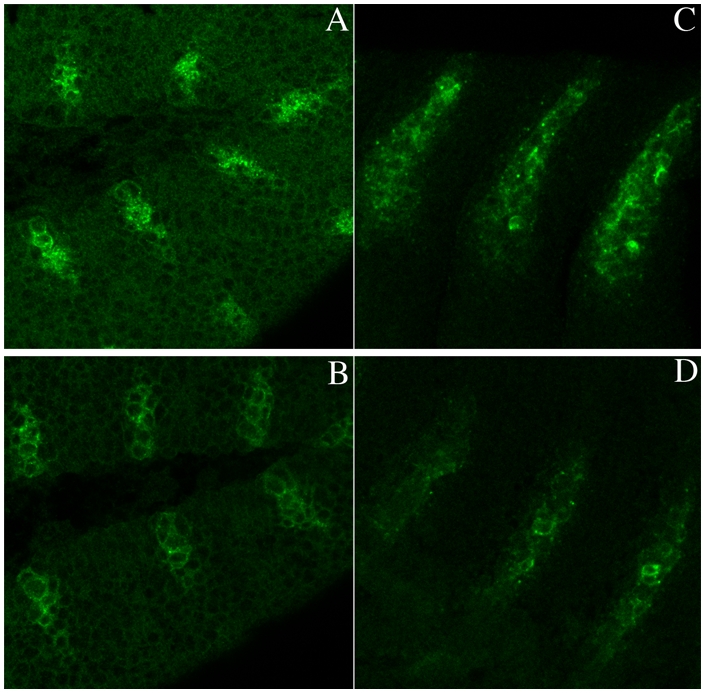
Reduced *wingless* expression in embryos compromised for *gγ1*. Panels A and B: Embryos from *gγ1^N159^/ Cy0, en:LacZ* stock were collected and fixed using standard procedures. Embryos were genotyped by probing with β-galactosidase antibodies (imaged in red: not shown), while Wg accumulation was visualized by probing with Wg (imaged in green) antibodies. Embryos carrying *en:LacZ* express β-galactosidase whereas homozygous mutant embryos do not. The embryo in panel A was positive for β-galactosidase (not shown) and has at least one wild type copy of *gγ1*. Note the high level of Wg accumulation in the stripes. The embryo in panel B β-galactosidase negative, and is homozygous for the *gγ1^N159^* mutation. Note the lower level of Wg expression. Panels C and D: Embryos from a *gγ1* ^k0817^ */Cyo, en:LacZ* stock were collected and fixed using standard procedures. As in panels A and B embryos were genotyped by probing with β-galactosidase antibodies (imaged in red: not shown), while Wg accumulation was visualized by probing with Wg (imaged in green) antibodies. The embryo in C is positive for β-galactosidase, while the embryo in D is not. Note the difference in Wg accumulation in the blow-up of three Wg stripes.

If *gγ1* functions downstream of *hmgcr* in the *hh* signaling pathway, then defects in *wg* expression should also be evident in *gγ1* mutant embryos. To determine if this is the case we compared *wg* expression in *gγ1^−^* embryos with their heterozygous *gγ1^−^/+* sibs. We found that *wg* expression in the homozygous mutant embryos is initially like wild type (or *gγ1^−^/+*); however as shown in Figure 2B for *gγ1^N159^* **,** the accumulation of Wg protein begins to decrease around stage 11–12 (compare the *gγ1^N159^* homozygote in panel B with the *gγ1^N159^*/+ sib control in panel A). Similar results were obtained for the *gγ1* ^k0817^ (compare panel C and D in Figure 1). The extent of reduction in Wg protein in the two *gγ1* mutants is not as severe as that seen in embryos compromised *hh*; however, as noted above this was also observed for *hmgcr* and likely reflects the perdurance of the maternally derived Gγ1.

Another gene whose expression in mid-embryogenesis depends upon *hh* signaling is *engrailed* (*en*). *en* is part of an autoregulatory loop that is established between the neighboring *hh* and *wg* expressing cells. *en* is transcribed in the *hh* expressing cells in response to the Wg ligand. When *wg* signaling is disrupted because of a reduction in *hh* signaling, *en* transcription is in turn downregulated. As would be expected from the effects of *gγ1* mutations on *wg* expression, we find that the accumulation of En protein is reduced in embryos homozygous mutant for both of the *gγ1* alleles compared to their wild type (*gγ1^−^/+*) sibs (see Figure S1).

### *Smo* Protein Is Mislocalized in *gγ1* Embryos

**Figure 3:**
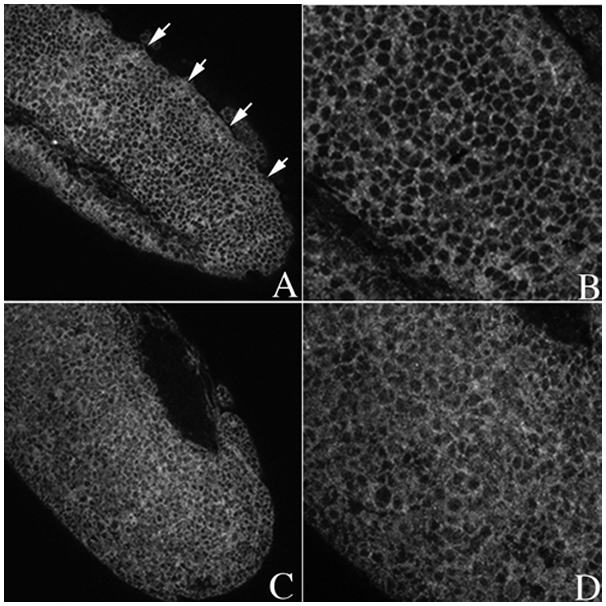
Localization of Smoothened receptor is altered in embryos compromised for *gγ1.* Embryos from *gγ1^N159^/ Cy0; en:LacZ* stock were collected, fixed using standard protocol and were subsequently identified by staining simultaneously with the β-galactosidase and Smo antibodies. Smo was imaged with the secondary antibodies coupled with Alexa 546. The figure shows stage 10–11 embryos. In the wild type control (*gγ1^N159^/ Cy0; en:LacZ* embryos) the intercellular distribution of Smo protein has a parasegmentally repeating pattern (see arrows in Panel A). In a ∼5 cell wide stripe across each parasegment Hh signaling leads to the relocalization of Smo protein to the membrane. In the remaining cells in each parasegment (∼5 cell wide stripe) Smo protein remains largely cytoplasmic. This can be seen in the magnified view in Panel B. In *gγ1^−^* embryos, this parasegmentally repeating pattern of Smo protein localization is largely lost (see Panel C). In most cells in each parasgement the Smo protein remains diffusely distributed through cytoplasm (see Panel D).

While the effects of *gγ1* mutations on *wg* and *en* expression would be consistent with a role in *hh* signaling, it is also possible that *gγ1* activity is required at some other point in the *hh-wg* autoregulatory loop, for example, in the expression of the Wg or En proteins. For this reason we next examined the effects of *gγ1* on the Smo receptor which is a more direct target for the Hh ligand in the receiving cells. Upon reception of the Hh signal the Smo receptor is relocalized from intracellular membrane vesicles to the cell surface [29],[30]. When *hh* signaling is compromised, this relocalization does not occur, and the Smo protein remains predominantly cytoplasmic in the receiving cells. Since Smo is not properly relocalized in *hmgcr* mutant embryos, a similar defect would be expected in *gγ1* mutants if *gγ1* functions downstream of *hmgcr* in the *hh* signaling pathway. Figure 3 shows that this prediction holds. In this experiment we compared the localization of the Smo receptor in homozygous *gγ1* mutant embryos with their heterozygous sibs. The pattern of Smo accumulation in the heterozygous *gγ1^−^/+* embryos (panels A and B) resembles wild type. There are a series of stripes that are approximately 5 cells wide in which the Smo protein is largely localized to the plasma membrane. These stripes are separated from each other by an equivalent band of about 5 cells that have a lower level of Smo at the surface of the cell. In homozygous *gγ1* mutant embryos this Smo distribution pattern is disrupted. Although a weak stripe pattern can still be discerned in the homozygous mutant embryos (see panel C) it is much less distinct than in the heteterozygous sibs (panel A). Panel D shows that Smo remains largely cytoplasmic in most of the cells in each segment and is not tightly localized at the plasma membrane as it is in wild type or *gγ1^−^/+* heterozygous embryos.

### Embryos Compromised for *gγ1* Display an Altered *Hh* Protein Distribution

**Figure 4:**
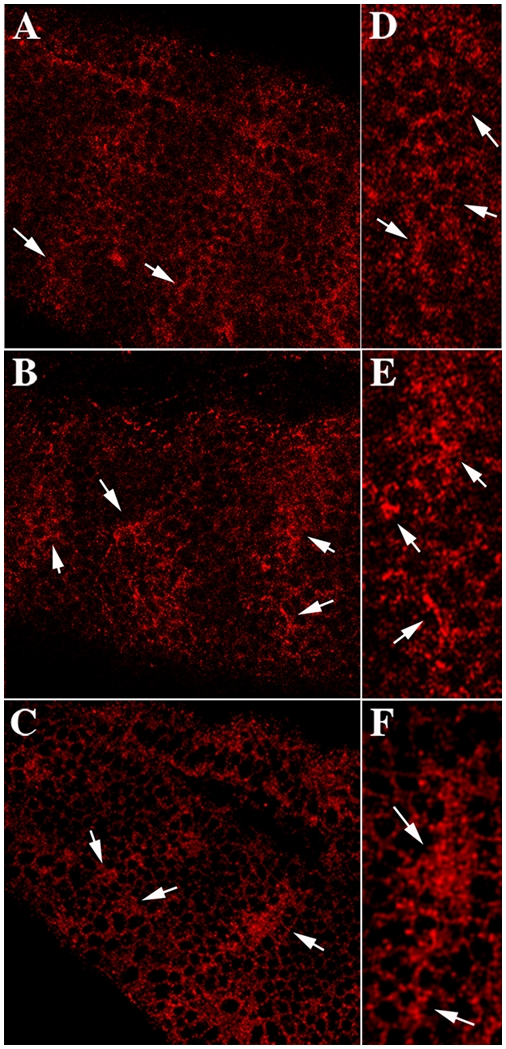
Spread of Hh ligand is restricted in *gγ1^−^* embryos. Embryos from *gγ1^N159^/ Cy0; en:LacZ* stock were collected, fixed using standard protocol and were subsequently identified by staining simultaneously with the β-galactosidase and anti-Hh antibodies. Hh specific signal was imaged with the secondary antibodies coupled to Alexa 546 (Red). The figure shows stage 10–11 wild type (panel A) and *gγ1^N159^* (panels B and C) embryos. In wild type embryos, two rows of cells per segment express Hh protein. In these cells Hh protein is distributed around the membrane in a grainy or punctate pattern (see arrowheads). The Hh ligand is released and it spreads in a graded fashion through the parasegment. In the *gγ1^N159^* embryos (panels B and C) Hh is not properly released from *hh* expressing cells and high levels of Hh protein accumulate in these cells. The grainy pattern of Hh protein around the cell membrane is less evident and instead Hh protein accumulates in larger clumps or aggregates that appear to be distributed in the cytoplasm rather than just at the membrane (see arrows). Panels D, E and F are enlargements of the Hh stripe in the embryos shown in panels A, B and C respectively. Arrows in panel D show the grainy punctate pattern of Hh protein in wild type embryos (the LPS), while the arrows in panels E and F show the larger clumps or aggregates of Hh protein that accumulate in cells of mutant embryos.

The finding that Smo protein does not properly relocalize in *gγ1* mutant embryos would be consistent with the idea that Gγ1 acts downstream of *hmgcr* to promote the efficient release and/or transport of Hh protein. To test this hypothesis further, we compared the pattern of Hh accumulation in *gγ1* mutant embryos with their heterozygous sibs. The distribution of Hh protein in *gγ1^−^/+* embryos resembles that seen in wild type [22]–[26]. Hh is expressed in each parasegment in a two cell wide stripe, and most of the protein in these Hh expressing cells is distributed in the cell membrane in a fine grain or punctate pattern (see arrowheads in panel A of Figure 4). Emanating in both directions from the two cell wide stripe is an Hh protein gradient that appears to extend through much of the parasegment. In this gradient the highest levels of Hh protein are observed associated with cells adjacent to the two Hh expressing cells, while lower levels of protein are found in more distant cells. The distribution of Hh protein in *gγ1* mutant embryos (panels B and C) resembles that seen in *hmgcr* mutant embryos [14]. First, in spite of the fact that the overall level of Hh expression is expected to be reduced in these embryos because of the disruption in the *wg-hh* positive autoregulatory loop (see above), the relative amount of Hh in cells in the *hh* stripes appears higher than in wild type embryos, while there is a concomitant reduction in the amount of Hh in the gradient that extends through the interstripe region (compare panels A with B & C). Second, the normal grainy or punctate pattern of Hh protein localized around the basolateral membrane of the *hh* expressing cells that is seen in wild type embryos (see arrows in panel A and in the enlargement in panel D) is largely lost. Instead, Hh accumulates in larger “clumps” or aggregates (see arrows in panels B and C and in the enlargement in panels E and F) that in many instances seem to be displaced from the cell membranes (see top arrows in panel E and F).

### *qm* Is Required To Promote the Release/Transmission of the *Hh* Ligand

**Figure 5:**
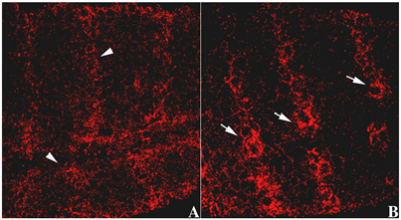
Distribution of Hh ligand is altered in *qm* embryos. Embryos from *qm^−^/ Cy0, ftz-LacZ* stock were collected, fixed using standard protocol and were subsequently identified by staining simultaneously with the β-galactosidase (not shown) and Hh antibodies (imaged in red). Panel A: Wild type control (*qm^−^/Cy0, ftz-LacZ*). Panel B: *qm^−^* embryo. In the control embryos, Hh protein synthesized in two rows of cells per parasegment is released and spreads through the segment. Within the Hh expressing cells, Hh protein is localized around the membrane in a grainy or punctate pattern (see arrowheads in Panel A). In *qm* the release/transmission of the Hh protein is abnormal. The level of Hh in the interstripe region is considerably diminished suggesting that like *gγ1*, the *qm* gene is required for the efficient release and/or transport of the Hh protein. Consistent with this suggestion, Hh protein appears to accumulate in the Hh expressing cells. Like *gγ1*, the distribution of Hh in the expressing cells is abnormal. Instead of the characteristic grainy or punctate pattern of Hh protein localized around the cell membranes, Hh accumulates in clumps or aggregates (see arrows).

The results described in the previous sections demonstrate that like *hmgcr*, g*γ1* is required for the efficient release/transmission of the Hh ligand by *hh* producing cells. Since the role of the isoprenoid branch of the *hmgcr* mevalonate pathway in heart development is the geranylgeranylation of Gγ1, a plausible idea is that the function of *hmgcr* in *hh* signaling is to provide substrates for the modification of the Gγ1 protein. If this model is correct, then gene products that are downstream of *hmgcr* in the geranylgeranylation pathway should also be required for the release/transmission of the Hh ligand. To test this prediction we examined the distribution of Hh protein in *qm* mutant embryos. As described above, *qm* encodes geranylgeranyl diphosphate synthetase and this enzyme produces the substrate, GGPP, that is used by the geranyl transferase to modify Gγ1. Figure 5 shows the distribution of Hh in a homozygous *qm* mutant embryo (panel B) and its heterozygous *qm^−^/+*sibs (panel A). As observed for both *hmgcr* [14] and *gγ1* (see above), the Hh ligand is inappropriately retained in the *hh* producing cells in the *qm* mutant embryos (compare Hh distribution in panels A and B). Like *gγ1* the characteristic punctate distribution of Hh protein around the membranes of *hh* expressing cells (arrowheads in pane A) is reduced or lost and instead Hh accumulates in clumps or large aggregates (arrows in panel B). It should also be noted that this particular *qm* mutation appears to cause a more pronounced defect in the release/transmission of the Hh ligand than is observed for *gγ1* (compare Figures 4 and 5), while the defects in Hh distribution evident in *hmgcr* mutant embryos [14] are roughly intermediate between that in *qm* and *gγ1*.

### Embryos Compromised for *gγ1* Activity Display Germ Cell Migration Defects

The findings describe above indicate that *gγ1* represents an endpoint for the isoprenoid branch of the *hmgcr* mevalonate pathway in the *hh* signaling pathway, and that like *hmgcr*, *gγ1* is required for the efficient release/transmission of the Hh ligand. Since several components of this *hmgcr* mevalonate→isoprenoid pathway have also been implicated in the production or transmission of the germ cell attractant by the SGPs [15], we tested whether mutations in *gγ1* have any effects on germ cell migration. Embryos collected from *gγ1* ^k0817^ and *gγ1^N159^* stocks carrying an *en:LacZ* marked 2^nd^ chromosome balancer were stained with b-galactosidase antibodies to identify the homozygous mutant embryos and Vasa antibodies to visualize the germ cells.

**Figure 6:**
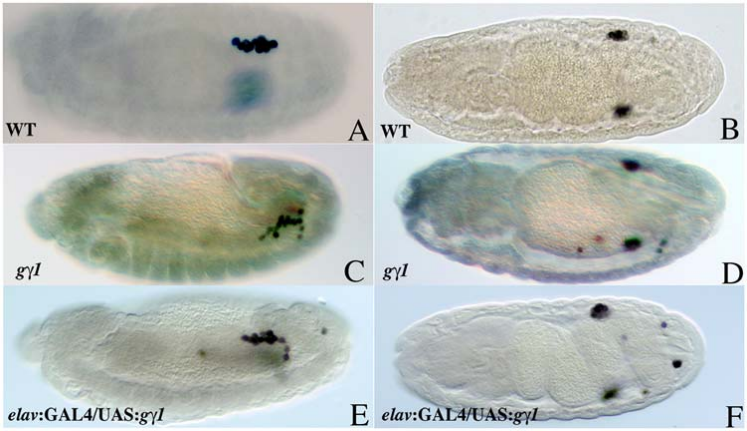
Either ‘loss’ or ‘gain’ of *gγ1* function leads to germ cell migration defects. Embryos from either the *gγ1^−^/ Cy0, en:LacZ* stock or from *UAS-gγ1* X *elav-GAL4* cross were collected and fixed using standard histochemical technique. Wild type embryos derived from Oregon R stock were used as control (Panels A and B). Embryos from the *gγ1^−^/ Cy0, en:LacZ* stock were identified by simultaneously staining them with b-galactosidase antibody (not shown). Germ cell migration was assessed using anti-Vasa antibodies. A: Wild type stage 13 embryo. B: Wild type stage 15 embryo. C: *gγ1^N159^* stage 13 embryo. D: *gγ1^N159^* stage 15 embryo. E: *UAS-gγ1/ elav-GAL4* stage 15 embryo. F: *UAS-gγ1/ elav-GAL4* stage 15 embryo.

In wild type embryos (or in *gγ1^−^/Cyo en:LacZ* embryos) germ cells associate with the SGPs in parasegments 10–13 at stages 12–13 (see stage 13 WT embryo in Figure 6A) and the two cell types coalesce into the embryonic gonad at stages 14–15 (see stage 15 WT embryo Figure 6B). Although germ cells that fail to coalesce into the embryonic gonad are sometimes seen in wild type embryos, the number of lost germ cells is generally rather low. The germ cells in *gγ1^−^* embryos appear to have no difficulty in exiting the midgut pocket at stage 9–10, while movement along the surface of the midgut also appears to be comparatively normal. However, as illustrated in panel C of Figure 6, defects in migration are clearly evident by stage 13. In this embryo, several of the germ cells are not properly aligned with the SGPs in PS10–13 (compare with wild type in panel A). This problem persists and in stage 15 *gγ1^−^* embryos germ cells that haven't coalesced into the embryonic gonad can be seen scattered in the posterior (see panel D). Quantitation indicates that in wild type the vast majority (90%) of the stage 15 embryos (n = 20) have few if any (0 to 2) scattered germ cells. In contrast, about 36% of the *gγ1^1N159^* embryos (n = 20) have 3 to 4 scattered germ cells while nearly 40% have 5 or more scattered germ cells. Similar results were obtained for the second *gγ1* allele, *gγ1* ^k0817^ (see Figure S2). Though the severity of the germ cell migration defects in the two *gγ1^−^* mutants is similar to that reported for embryos zygotically compromised for either *fpps* or *qm*, it is not as strong as that observed for *hmgcr* mutant embryos [9], or for embryos that lack both zygotic and maternal (*m^−^ z^−^*) *fpps* [15]. While compromising both maternal and zygotic *gγ1* would likely increase the severity of the germ cell migration defects as seen for *fpps*, the very severe patterning abnormalities observed in *m^−^z^−^* embryos [28] would make effects on germ cell migration impossible to interpret. These findings indicate that *gγ1* is involved in germ cell migration just like the three enzymes, *fpps*, *qm* and *β-GGT1* that are downstream of *hmgcr* in the geranylgeranylation branch of the mevalonate pathway.

### Ectopic Expression of *Gγ1* Induces Germ Cell Migration Defects

Ectopic expression of *hmgcr*, *qm* or *fpps* can induce the production of the germ cell attractant in inappropriate tissues. This ectopic source of attractant competes with the attractant produced by the SGPs and confuses the germ cells, disrupting their migration towards the SGPs [9],[15]. If *gγ1* is functioning in the same pathway as these three enzymes, then it should also be possible to confuse germ cells by ectopically expressing *gγ1*. To test this hypothesis, females carrying the CNS driver *elav-GAL4* were mated to males carrying a UAS transgene that drives a flag-tagged Gγ1 protein and the resulting *elav-GAL4/UAS-flag gγ1* embryos were stained with Vasa antibodies to mark the germ cells.

Figure 6E and 6F show that misexpression of Gγ1 in the central nervous system leads to a weak but reproducible germ cell migration defect. In wild type 90% of the stage 14–15 embryos have 0–2 scattered germ cells, while about 10% have 3 or more scattered or lost germ cells. In contrast in *elav-GAL4/UAS-flag gγ1* embryos (n = 51), more than 40% of the embryos have 3 or more lost germ cells. The effects of *elav* driven Gγ1 expression are less than that reported for *elav* driven Hgmcr expresson (100% have 3 or more scattered germ cells) but equivalent to that observed for *elav* dependent misexpression of either Fpps or Qm (approximately 40% with 3 or more scattered germ cells: see 15).

### Ectopic Expression of *gγ1* in *hh* Producing Cells Induces Aberrant Germ Cell Migration

**Figure 7:**
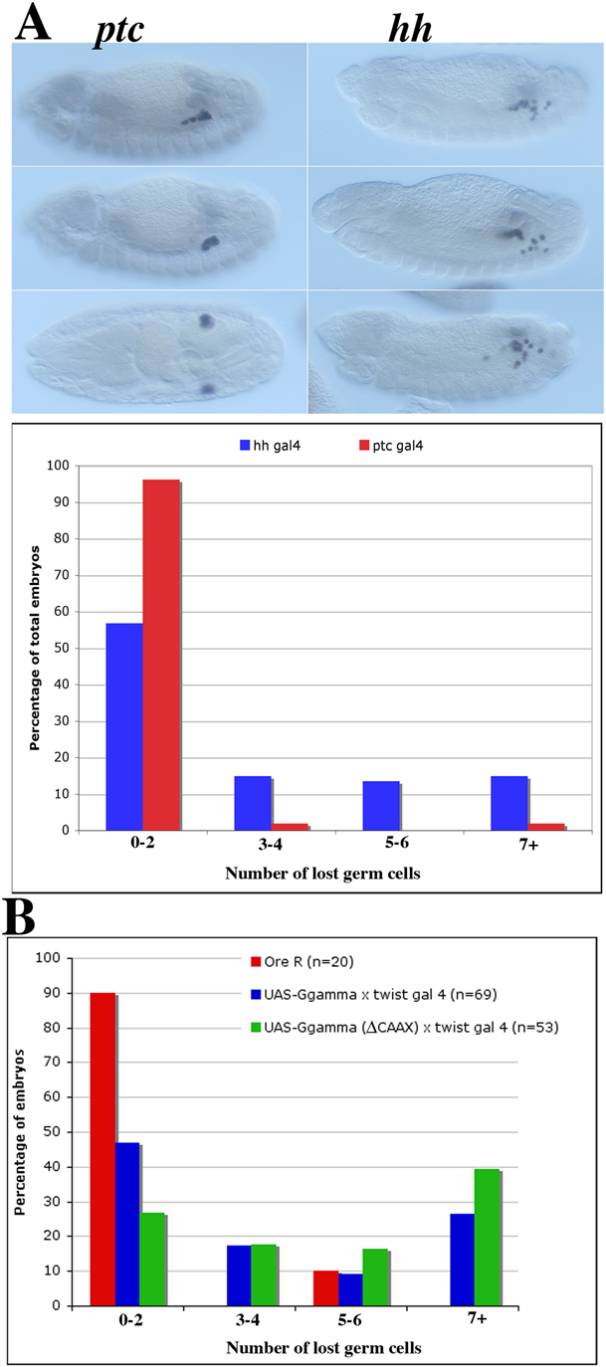
Manipulations of *gγ1* activity disrupt germ cell migration. (A) Ectopic expression of *gγ1* in *hh* producing cells not in *hh* receiving cells can induce germ cell migration defects. Whole mount staining of stage 13–15 embryos with antibodies against Vasa protein. Females carrying two copies of *UAS-gγ1* were mated independently either with the *ptc-GAL4/ptc-GAL4* males (panels on left) or *hh*-*GAL4/TM6 Ubx-LacZ* males (panels on right). Embryos (10–14 hr old) were collected, fixed and then probed with β-galactosidase and Vasa antibodies. In the case of *hh*-*GAL4* driver, embryos of the correct genotype were identified by the absence of β-galactosidase. The staining was visualized using standard immunohistochemical techniques. As can be seen by the comparison of the panels, *gγ1* is able to induce germ cell migration defects only when it is overexpressed using *hh*-*GAL4* whereas *gγ1* overexpression using *ptc-GAL4* leads to essentially wild type germ cell migration. As shown in the bar-diagram in the lower half of A, more than 90% of the *ptc-GAL4/UAS-gγ1* embryos (represented with red colored bars) display less than 2 lost germ cells whereas close to 30% of the *hh-GAL4/UAS-gγ1* embryos (represented with blue colored bars) have more than 5 lost germ cells. *hh-GAL4/UAS-gγ1* (n = 74), *ptc-GAL4/UAS-gγ1* (n = 53). (B) Ectopic expression of *gγ1* and *gγ1-ΔCAAX* in the mesoderm induces germ cell migration defects. In this experiment embryos produced by females carrying the *UAS:gγ1* or *UAS gγ1-ΔCAAX* mated to *twist* GAL4 males were stained with Vasa antibody and the number of lost or scattered germ cells in each embryo was counted. As shown in the bar graph, ectopic expression of either Gγ1 or Gγ1-ΔCAAX using the *twist* driver induced germ cell migration defects. As explained in the text, these defects likely arise for different reasons. Ore R (n =  20), *UAS-gγ1* (N = 69), *UAS- gγ1-ΔCAAX* (n = 53).

In previous studies we found that expression of Hmgcr protein in *hh* producing cells was much more effective in inducing aberrant germ cell migration than when it was expressed in *hh* receiving cells. If *gγ1* functions downstream of *hmgcr* in the production of the germ cell attractant, then ectopic Gγ1 should also have a more pronounced effect on germ cell migration when it is expressed in *hh* producing cells then when it is expressed in *hh* receiving cells. The experiment in Figure 7A shows that this prediction holds. There is little or no effect on germ cell migration when Gγ1 expression is induced in *hh* receiving cells by a *ptc-GAL4* driver. In this case, less than 10% of the embryos have 3 or more lost germ cells, which is comparable to that seen in wild type embryos (see bar graph in Figure 7A). In contrast, nearly one half of the embryos have 3 or more lost germ cells when Gγ1 is expressed in *hh* producing cells under the control of *hh-GAL4* driver (Figure 7A). While this result indicates that like Hmgcr, Gγ1 must be misexpressed in *hh* producing cells in order to induce aberrant germ cell migration, it is important to note that the effects of ectopic Gγ1 are less severe than that produced when Hmgcr expression is driven by the same *hh-GAL4* driver [10]. Consistent with this difference, we do not observe any obvious alteration in the parasegmental distribution of Hh protein in *UAS-gγ1/hh-GAL4* embryos (not shown). By contrast, substantially more Hh protein is found in the interstripe regions when Hmgcr expression is driven by *hh-GAL4* in *hh* producing cells [10].

### Synergistic Genetic Interactions between *gγ1* and Either *hmgcr* or *hh* Disrupt Germ Cell Migration

While there are few if any defects in germ cell migration in *hmgcr^−^/+* embryos, synergistic interactions are observed when *hmgcr^−^* is combined with mutations in two components of the *hh* signaling pathway *hh* and *disp* [10]. The perturbations in germ cell migration observed in the *trans*-heterozygotes taken together with the effects of *hmgcr* on the release/transmission of the Hh ligand from *hh* producing cells lent support to the hypothesis that the primary function of *hmgcr* in the production of the germ cell attractant by the SGPs is to potentiate the Hh signal emanating from these cells. Since the results presented above suggest that *gγ1* also functions in the release/transmission of the Hh ligand, we wondered whether equivalent synergistic genetic interactions would also be observed for *gγ1*.

**Figure 8:**
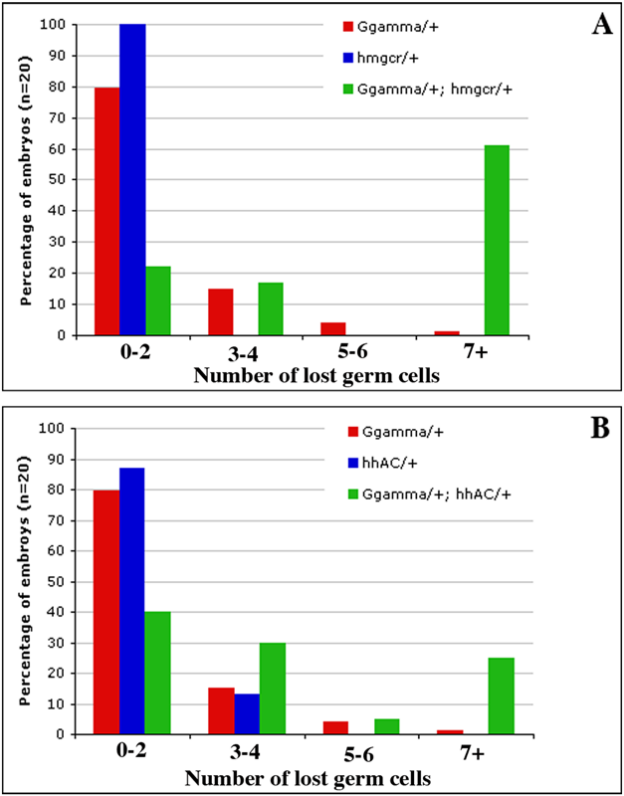
Germ cell migration defects induced by partial loss of *gγ1* are enhanced further by reducing either *hmgcr or hh* activity. Embryos between stages 12–15 of the indicated genotype were stained with anti-Vasa and β-galactosidase antibody and staining was visualized with standard immunohistochemcial techniques. Total number of germ cells that failed to associate with SGPs and remained scattered were counted per embryo. 25 embryos of each genotype were analyzed. Top panel shows that when embryos are heterozygous either for *gγ1* or *hmgcr*, more than 80% of the embryos display 0–2 lost germ cells (blue and red bars respectively). But when embryos are simultaneously compromised for both *gγ1* and *hmgcr*, more than 60% of the embryos have 7 or more lost germ cells (yellow bars). Bottom panel shows similar synergistic interaction between *gγ1* or *hmgcr*. Although the enhancement in germ cell migration defects is less severe compared to that seen with *hmgcr* (30% of the total number of embryos of the genotype *gγ1/+; hh/+* show more than 7 lost germ cells), the germ cell migration defects in embryos simultaneously compromised for *gγ1* and *hh* are clearly more severe as opposed to either *gγ1/+* or *hh/+* embryos.

We first tested for interactions between *gγ1* and *hmgcr*. Like *hmgcr*, there are at most only very modest defects in germ cell migration in *gγ1^N159^/+* embryos. However, more than 60% of the *trans*-heterozygous embryos have 7 or more lost germ cells (see Figure 8A). Next we tested for genetic interactions between *gγ1^N159^*/+ and *hh*. As shown in Figure 8B, the minor germ cell migration defects observed in *hh^−^/+* embryos are greatly enhanced when the *hh* mutation is combined with *gγ1^N159^*. These results support the idea that *gγ1* could function in the germ cell migration pathway by facilitating the release/transmission of the Hh ligand.

### Misexpression of the *gγ1* CAAX Deletion Mutant Induces Germ Cell Migration Defects

The results described in the previous sections suggest that the *hmgcr* mevalonate pathway is required in germ cell migration because g*γ1* must be geranylgeranylated in order for it to potentiate Hh signaling by the SGPs. To test this idea further, we examined the effects of misexpressing either the wild type Gγ1 or Gγ1 proteins that have mutations in the C-terminal CTVL geranylgeranylation motif in the mesoderm using a *twist-GAL4* driver. We anticipated that misexpressing wild type Gγ1 using the *twist* driver would induce aberrant germ cell migration because it would inappropriately potentiate signaling by *hh* expressing cells elsewhere in the mesoderm such as the fat body precursor cells (FBP). The *hh* signal emanating from these cells would compete with the signal from the SGPs, and this would confuse the migrating germ cells. The results shown in Figure 7B indicate that this expectation is met. While there are only a few wild type embryos in this experiment which have more than 2 lost germ cells, more than half of the *twist-GAL4:UAS-*g*γ1* embyros have at least 3 lost or mismigrated germ cells.

We also anticipated that misexpressing Gγ1 proteins that have mutations in the C-terminal geranylgeranylation motif would induce germ cell migration defects as well. Gγ1 forms a heterodimer with a second G protein Gβ and together these two proteins interact with a third G protein, Gα to form a heterotrimeric complex. In order to form a functional complex with Gα and also interact with other factors and effectors, the Gβ:Gγ1 heterodimer must be anchored to the membrane and this is thought to be dependent upon geranylgeranylation of the Gγ1 protein [31],[32]. We reasoned that Gγ1 mutant proteins that cannot be geranylated would likely behave as dominant negatives because they would compete with the endogenous Gγ1 protein for complex formation with Gβ, and thus reduce the effective concentration of functional membrane bound Gγ1:Gβ heterodimers. This idea is support by studies on the *Drosophila* eye specific Gγ protein Gγe. The C-terminal sequence Gγe is C-V-I-M which corresponds to the signal for farnesylation rather than geranylgeranylation. Mutations in Gγe that eliminate farnesylation have no effect on the formation of Gγe:Gβe heterodimers; however, these heterodimers do not interact with the membrane and are non-functional [32]. When the mutant Gγe protein is overexpressed it competes with the endogenous Gγe protein for heterodimer formation with Gβe, reducing the amount of functional membrane associated Gγe:Gβe heterodimers and disrupting signal transduction. If gernaylation defective Gγ1 proteins also behave like dominant negatives, they would be expected to interfere with the efficient release of the germ cell attractant by the SGPs when they are ectopically expressed in mesodermal cells and this should perturb germ cell migration. We tested two different Gγ1 mutant proteins, one in which the C-terminal CTVL motif is deleted (Gγ1-ΔCAAX) and the other in which the geranylated Cys residue is replaced by Ser (Gγ1-C67S) [16]. As shown in Figure 7B and Figure S3, ectopic expression of the Gγ1-ΔCAAX protein disrupts germ cell migration and about 75% of the *twist-GAL4/UAS-*g*γ1-ΔCAAX* transgene embryos have 3 or more lost germ cells. (Note: Figure S3 shows that ectopic expression of Gγ1-ΔCAAX in germ cells also disrupts their migration.) With the caveat that there may be differences in expression levels of the *UAS* transgene, it would appear that the germ cell migration defects induced by the geranylation defective Gγ1-ΔCAAX protein are somewhat more pronounced than those observed with wild type Gγ1. Consistent with this possibility, the Gγ1-C67S mutant protein also induces more extensive germ cell migration defects than wild type (not shown).

## Discussion

### *Gγ1* Is Required for *Hh* Signaling

Hh functions as an instructive cue in many different biological contexts. The signaling molecule is secreted from *hh* expressing cells and it induces morphogenesis in a concentration dependent fashion in neighboring cells by regulating the transcription of downstream target genes. Several mechanisms control the range and inductive activity of the Hh protein. These include the autoprocessing and lipidation [33]–[35]. Hh has two different lipid modifications that are important for the proper functioning of the Hh ligand. One is the palmitoylation of the N terminus which seems to be critical for signaling activity, while the other is the addition of cholesterol to the C terminus. The C-terminal cholesterol moiety is thought to be important for the dimerization of the Hh protein and for its assembly into LPSs (Large Punctate Particles) prior to secretion [36]–[39]. The LPSs appear to be lipid vesicles or micelles and they are thought to provide a hydrophobic environment for the lipid modified Hh which facilitates its movement through the extracellular matrix after it is secreted. The release and subsequent transport of the Hh ligand also requires specialized proteins that function in either Hh producing cells or in cells/compartments that are destined to receive the Hh ligand. The transporter class protein, Disp, and a secreted protein Shifted (Shf) are required in *hh* expressing cells for the efficient release and transmission of the Hh ligand [25], [40]–[42]. In *shf* mutants, the basolateral accumulation of Hh protein in the wing disc is disrupted, while apical accumulation appears to be normal. The subsequent transport of the Hh ligand to the receiving cells depends upon the glypicans Dally-like (Dlp) and Dally, which are components of the extracellular matrix, and enzymes that are needed for glycosaminoglycan biosynthesis namely Sulfateless and Tout-velu [43]–[45]. The glycosaminoglycan is thought to promote long range signaling by Hh and other signaling molecules such as Wg by passing the ligand from one cell to its neighbor instead of presenting the ligand to the receptor [46]. It is thought to do so by directing the ligands to the lateral membranes where endocytosis is less efficient [47].

There are likely to be an extensive array of accessory factors like *disp* and *dally* that are required for the efficient release of the Hh ligand from *hh* expressing cells and its subsequent transport or transmission from one neighboring receiving cell to the next. We have previously shown that one such gene encodes the mevalonate biosynthetic enzyme Hmgcr [14]. We found that *hmgcr* is required in Hh expressing cells to facilitate the release or transmission of the Hh ligand; however, it was not clear from our studies why the biosynthesis of mevalonate would be important for the release/transmission of the Hh ligand in flies. The obvious explanation, that it is required for the synthesis of the cholesterol that is used to modify Hh, was not likely to be correct as flies do not have the downstream enzymes for cholesterol biosynthesis [15]. In the work reported here we have resolved this question. We show that the downstream target for *hmgcr* in the *hh* signaling pathway is the heterotrimeric G protein, Gγ1, which must be geranylated in order to be active [17],[31],[48]. Like *hmgcr* and other genes that are required to promote *hh* signaling, mutations in *gγ1* dominantly suppress the gain-of-function wing phenotypes of *hh^Mrt^* in adult flies. In the embryo, the expression of *wg* which is activated by *hh* in the receiving cells is downregulated in both *hmgcr* and *gγ1* mutants. This is also true for the *en* gene which is normally activated by *wg* signaling in *hh* expressing cells as part of the autoregulatory circuit that sustains *hh* and *wg* expression as the embryo develops. These transcriptional defects arise because the Hh signal is not properly conveyed to *hh* receiving cells. In wild type embryos Smo protein is redistributed to the membranes of the receiving cells when they receive the Hh signal transmitted from the neighboring Hh producing cells. As observed for *hmgcr*, Smo protein is not correctly relocalized in *gγ1* mutant embryos, and instead it remains largely cytoplasmic. Finally, in the ectoderm of wild type embryos there is a gradient of Hh protein extending into the parasegment from the two cell wide stripe of *hh* expressing cells. Like *hmgcr*, this gradient is not properly formed in *gγ1* mutant embryos, and instead Hh protein is inappropriately retained in the Hh producing cells.

Since isoprenoid modifications, either farnesylation or geranylation, are known to be critical for the functioning of the Gγ family of proteins, these observations would argue that *hmgcr* is required for the release of the Hh ligand because it provides a precursor that is needed for the geranylgeranylation of Gγ1. This conclusion is supported by the finding that *qm*, which synthesizes the activated substrate, GGPP, that is used to geranylate Gγ1, is also required for the release of the Hh ligand from *hh* expressing cells. While these results implicate the biosynthetic pathway leading from mevalonate to the geranylation of Gγ1 in the proper release of the Hh ligand, we cannot exclude the possibility that there are important targets for geranylation in addition to Gγ1 or that other products of mevalonate might play some role in the *hh* signaling pathway. These possibilities remain open for a number of reasons.

First, the defects in the release of Hh observed in antibody staining experiments seem to be more severe in the *qm* mutant (and to a lesser extent in *hmgcr*: see 14) than in the *gγ1* mutants we examined. One explanation for this difference is that the Qm enzymatic product, GGPP, is used for the geranylation of other proteins that are important for the release of the Hh ligand. However, this could also be due to, for example, differences in the perdurance of the maternal Qm and Gγ1 proteins.

Second, ectopic expression of Hmgcr in *hh* expressing cells causes a readily discernible change in Hh protein distribution across the parasegment and relatively high levels of Hh are found even near the middle of the interstripe region. By contrast, we could not detect an equivalent alteration in Hh distribution when Gγ1 was ectopically expressed in *hh* producing (or receiving) cells. This difference could mean that the mevalonate produced by Hmgcr has uses in *hh* signaling besides the synthesis of GGPP and the geranylation of Gγ1.

An alternative and perhaps more interesting possibility is that the differences in the effects of misexpression on the release/transmission of Hh protein reflect the fact that Hmgcr is limiting whereas Gγ1 is not. Consistent with this idea, the distribution of *hmgcr* mRNAs becomes progressively restricted as development proceeds and by mid-embryogenesis (stages 10–15) *hmgcr* mRNAs are only detected in the SGPs [9]. By contrast, mRNAs encoding Gγ1, as well as several of the enzymes that are downstream of Hmgcr in the biosynthesis of GGPP, are much more widely expressed in the embryo at this stage [15],[28]. A possible consequence of this difference in mRNA distribution is that the levels of Hmgcr protein would increase dramatically when it is ectopically expressed in the ectoderm during mid-embryogenesis while this would not be true for Gγ1 or, for that matter, the other GGPP biosynthetic enzymes. The idea that Hmgcr is a limiting component of signaling pathways that depend upon geranylation Gγ1 (or other targets) is also supported by the defects in germ cell migration that are induced by ectopic expression of these proteins. For example, expression of *hmgcr* in the CNS cause much more severe abnormalities in germ cell migration than those observed when *fpps*, *qm,* or *gγ1* are misexpressed [15]. If these ideas were correct, than inducing or repressing the expression of the *hmgcr* gene would provide a novel posttranslational mechanism for regulating the potency of signaling molecules like Hh.

The effects of *gγ1*, *qm* and *hmgcr* on the distribution of Hh in the ectoderm indicates these genes are required for the release of the Hh ligand from *hh* expressing cells. For Gγ1, a role in releasing the Hh ligand from the sending cells would dovetail nicely with a recently discovered function of this G protein and its partners, Gβ and Gα in the transport of cargo from the *trans-*Golgi network (TGN) to the basolateral plasma membrane [19]–[21]. Since Hh protein appears to be specifically targeted to the basolateral membrane in punctate structures (LPSs) prior to secretion [22]–[25],[38],[39], it is not altogether surprising that components of the machinery needed for the transport of cargo from the TGN to the basolateral membrane would play a key a role in transmitting the Hh signal. Moreover, since Gγ1 requires geranylation for membrane association and activity, the retention of Hh in *qm* and *hmgcr* mutants would also be explained by a disruption in Gγ1-dependent TGN-plasma membrane transport. In this context it is interesting to note that while the levels of Wg are reduced in *hmgcr* [14] and *gγ1* mutants, there is no obvious over accumulation of the Wg protein inside *wg* expressing cells like that observed for Hh. That the *hmgcr→qm→gγ1* pathway would have no apparent effect on the release of Wg would make sense since this is thought to occur preferentially through the targeting of mRNAs to the apical surface of the cell [49].

Though the precise mechanisms for TGN-plasma membrane transport have yet to be elucidated, it is thought that the heterotrimeric G protein complexes mediate the release of cargo from the TGN by promoting membrane fission [25]. In one scenario, interaction of the cargo with an unidentified receptor in the TGN leads to the activation of the trimeric Gγ1:Gβ: Gα and the release of Gα The Gγ1:Gb heterodimer in turn activates several targets including phosphokinase C and a phosphoinostide-specific phospholipase C (PI-PLC) that generates diacylglycerol. PKC participates in cargo release from the TGN by activating Protein Kinase D (PKD) while locally high concentrations of diacylglycerol produced by PI-PLC are thought to change the properties of the TGN membranes and promote membrane fission. After membrane fission, the vesicle containing the cargo is then targeted to the basolateral plasma membrane [24],[25]. A requirement in the formation of cargo containing vesicles would fit well with the effects of *hmgcr*, *qm* and *gγ1* on the formation of the puncate Hh particles, or LPSs, that normally assemble along the basolateral membranes of Hh expressing cells. These LPSs are largely absent in *hmgcr*, *qm* and *gγ1* mutant embryos and instead Hh accumulates in much larger aggregates or clumps. While the precise origin of the LPSs is not known, they are thought to be lipid containing vesicles (or micelles) and it would be reasonable to think that they could be generated by the budding of Hh containing vesicles from the TGN. In this case, the large aggregates or clumps of Hh protein seen in *hmgcr*, *qm* and *gγ1* mutants would likely represent Hh trapped either in the TGN or in aberrant vesicles/structures that accumulate in the mutant cells when efficient cargo release from the TGN is disrupted.

While the idea that Gγ1 promotes the transport of Hh from the TGN to plasma membrane would seem to fit best with the known functions of Gγ1 and its collaborating G proteins, it is also possible that Gγ1 (plus Gβ and Gα) functions at earlier steps in the secretion pathway, for example. in the transport of Hh from Endoplasmic Reticulum to the Golgi [50]. Alternatively, it is possible that some novel activity of Gγ1 at the plasma membrane rather than in the TGN is needed. For example, it could function to prevent the newly formed LPSs from clumping together into larger aggregates. Further studies will be required to distinguish between these and other possible mechanisms.

### *Gγ1* Is Required for Germ Cell Migration

Studies by Santos and Lehmann [15] provided convincing evidence that *hmgcr* is required in the soma for germ cell migration because its biosynthetic product, mevalonate, is the precursor for the synthesis GGPP by Qm. They also found that GGPP is used in turn by geranylgeranyl transferse type 1 (*β-GGT1*) for the geranylation of some unknown target(s). The experiments presented here indicate that one (if not the only) somatic target in the germ cell migration pathway is Gγ1. Thus, the effects of both gain and loss of *gγ1* function on germ cell migration closely resemble those reported for *hmgcr, fpps, qm*, and *β-GGT1*. Also supporting the idea that Gγ1 must be a relevant target for the *hmgcr-*isoprenoid biosynthetic pathway, we find that Gγ1 proteins that cannot be geranylated behave as dominant negatives when ectopically expressed in the mesoderm and disrupt germ cell migration. In addition, there are other significant similarities between the two genes that have been studied in most detail, *gγ1* and *hmgcr*. First, both genes show synergistic genetic interactions with components of the *hh* signaling pathway that perturb the process of germ cell migration. Second, germ cell migration can be disrupted when *gγ1* or *hmgcr* are ectopically expressed in *hh* producing cells; however, there are no apparent effects when the genes are ectopically expressed in *hh* receiving cells.

Taken together with the fact that the SGPs are known to be a source of Hh these findings would argue that a critical function of the biosynthetic pathway leading from *hmgcr* to the geranylation of Gγ1 is to upregulate Hh signaling in the SGPs, and it is the Hh ligand produced by these cells that serves to attract the migrating germ cells. Importantly, this model accounts for a number of different observations. Since Hmgcr is expressed at high levels in the SGPs, but is not expressed elsewhere in the mesoderm, it would explain how Hh signaling could be specifically potentiated in a special sub-population of cells. It would also explain why the effects of *hmgcr* misexpression are much greater than misexpression of the other genes in the *hmgcr→gγ1* pathway that are more broadly transcribed in the embryo. Finally, it would explain why germ cells can be misdirected by ectopic expression of *hh*, *hmgcr*, the downstream genes in the geranylation biosynthetic pathway, and *gγ1* in a variety of different tissues. By contrast, if the SGPs were to induce germ cell migration by expressing some unique and dedicated *hmgcr→gγ1* dependent attractant, it is hard to understand how misexpression of these different upstream genes would be able orchestrate the production of this special molecule in a variety of cells and tissues that have little resemblance to the SGPs. It should be noted, however, that our results would also be compatible with more complicated models. For example, it is possible that the potentiation of Hh signaling by the *hmgcr→gγ1* pathway induces the production of a specialized and as yet unknown germ cell attractant. Likewise, we also can not exclude the possibility that there is some other target for geranylation besides Gγ1 which is important for the production or activity of a second germ cell attractant and that this unknown molecule functions in concert with Hh to direct germ cell migration towards the SGPs. However, in either of these more complicated scenarios, the unknown germ cell attractant would have to be a molecule that can be induced in many different cell types in the embryo, but apparently only if these cells also express the Hh protein.

## Materials and Methods

### Immunohistochemistry

The embryo stainings were performed essentially as described in 51. Vasa (from Paul Lasko) and Hh (from Tom Kornberg) antibodies are rabbit polyclonal antibodies. Both were used at a 1∶500 dilution. Engrailed and Wingless antibodies are mouse monoclonal antibodies and were used at 1;10 dilution. β-Galactosidase antibody was either a rabbit polyclonal purchased from Kappel (used at 1∶1000 dilution) or a mouse monoclonal antibody from Developmental Hybridoma Bank (used at 1∶10 dilution). Smoothened antibody (anti-rat) was a kind gift from Steve Cohen and was used at 1∶500 dilution. For confocal analysis a magnification of 40× was used in almost all the instances and images were collected using identical settings for the control and experimental samples. Multiple pairs of wild type (sibs) and mutant embryos were imaged in each case and representative examples are presented.

### Mutant and Misexpression Analysis

*gγ1* mutant stocks, *gγ1^N159^* and *gγ1* ^k0817^, were obtained from Fumio Matsuzaki while the various 1UAS- *gγ1* stocks (*gγ1*, *gγ1 ΔCAAX* and *gγ1 C67S*) were kindly provided by the Olson lab. The other UAS and GAL4 stocks used for the misexpression studies: *UAS- hmgcr, hairy-*GAL4, *elav-*GAL4, *nanos-*GAL4, *patched-*GAL4, UAS*-β-galactosidase*, *hh-*GAL4*/TM6 Ubx-LacZ*. In most experiments, males carrying two of the copies UAS transgene were mated with virgin females carrying two copies of the GAL4 transgene. The resulting progeny embryos were fixed and stained for subsequent analysis [51].

## Supporting Information

Figure S1 Engrailed expression is not properly maintained in *gγ1* mutant embryos. Embryos from the *gγ1^N159^/ Cy0, en:LacZ* stock were collected and fixed using standard procedure. Embryos were genotyped by simultaneously staining them with β-galactosidase (imaged in green: not shown) and En (imaged in red) antibodies. Balancer embryos (Panel A) show strong En specific expression in 14 stripes. By contrast, En specific signal starts to decline by Stage 11 in the homozygous *gγ1^N159^* embryos (Panels B and C). As illustrated in these two panels there is some variation in the extent of the reduction in En expression. In some embryos, moderate levels of En protein are detected (B) while in others only low levels are observed (C). Because of the variability in En accumulation in *gγ1^N159^* homozygous embryos, we classified the En staining pattern. For the heterozygous *gγ1^N159^/+* control, 6/7 embryos had high levels of En accumulation, while 1 embryo had a medium level of accumulation. For the homozygous *gγ1^N159^* embryos 4/11 (37%) had little En protein (like the example shown in the figure) while 4/11 had a medium level of En protein (like the example shown in the figure). The 3 remaining embryos (27%) resembled wild type. We also examined En expression in homozygous *gγ1* ^k0817^ embryos. In this experiment all of the heterozygous *gγ1* ^k0817^/+ control embryos had a high level of En protein (9 embryos). For the homozygous *gγ1* ^k0817^ mutant embryos 8/18 (44%) had a low level of En protein, while 5/18 (28%) had a medium level of En protein. Finally, 5/18 (28%) homozygous mutant embryos had a high level of En protein.
(3.2 MB TIF)

Figure S2 Germ cell migration defects are also observed in *gγ1* ^k0817^ mutant embryos. Embryos from a *gγ1* ^k0817^/*Cy0 en:LacZ* stock were probed with Vasa to mark the germ cells and β-galactosidase antibodies to identify heterozygous and homozygous mutant embryos. Panels A and B are *gγ1* ^k0817^/*Cy0 en:LacZ* embryos (note β-galactosidase expression) while panel C and D are mutant. Panel C shows embryo with 3 scattered cells whereas the embryo in panel D has more than 6 scattered germ cell cells. About 15% (4/22) of the mutant embryos had 3–4 scattered germ cells (example in panel C), while about 40% (8/22) of the mutant embryos had 5 or more scattered germ cells (example in panel D). The remaining embryos (10/22 or 45%) had 2 or fewer scattered germ cells.
(2.0 MB TIF)

Figure S3 Ectopic expression of *gγ1-ΔCAAX* in the mesoderm and in germ cells disrupts germ cell migration. Panels A–F show stage 13–15 *twist-GAL4/UAS*-*Gγ1-ΔCAAX* or *nos-GAL4/UAS*-*Gγ1-ΔCAAX* embryos probed with Vasa antibodies to visualize migrating germ cells. Panels A–D: Germ cell migration defects in *twist-GAL4/UAS*-*Gγ1-ΔCAAX* embryos. Panels E and F: Germ cell migration defects in *nos-GAL4/UAS*-*Gγ1-ΔCAAX* embryos. Recent studies by Kunwar et al. [52] on germ cell migration have suggested that *gγ1* has a cell autonomous requirement in germ cells. To test the cell autonomous function of *gγ1* in germ cell migration, these authors rescued the gastrulation defects of progeny from *gγ1* germline clone mothers using a *nullo-GAL4* to drive expression of a *UAS-gγ1* transgene. They reported that the pole cells in these embryos failed to migrate properly out of the midgut and exhibited phenotypes similar to those found for mutations in the G protein-coupled receptor (GPCR) gene *trapped in endoderm 1* (*tre1*). We wondered whether the geranylated form of Gγ1 is also required in the germline. To explore this possibility we ectopically expressed the dominant negative *gγ1* deletion mutant Gγ1-ΔCAAX in the germline. As shown in Figure S3E and S3F, ectopic expression of the Gγ1-ΔCAAX protein in germ cells using a *nos-GAL4* driver disrupts germ cell migration. We found that nearly 60% of the stage 13–15 *nos-GAL4/UAS*-*Gγ1-ΔCAAX* embryos had 3 or more lost germ cells, while 33% had 5 or more lost germ cells (n = 100 embryos). This is roughly equivalent to the germ cell migration defects evident when Gγ1-ΔCAAX is expressed in the mesoderm using a *twist-GAL4* driver. The effects of the dominant negative protein in germ cells would support the findings of Kunwar et al., and argue that *gγ1* (specifically geranylated Gγ1) has a cell autonomous function in these cells during their migration towards the SGPs. On the other hand, our results differ somewhat from those reported by Kunwar et al. in that we did not observe any obvious defects in the ability of the germ cells to exit the midgut when the Gγ1-ΔCAAX protein was expressed using the *nos-GAL4* driver. While there are a number of plausible reasons why a *tre1*-like phenotype wasn't observed, the most likely explanation is that not enough of the dominant negative Gγ1-ΔCAAX is generated to disrupt the *tre-1* dependent migration through the midgut epithelia. In particular, germ cells are known to be transcriptionally quiescent until just before they exit the midgut, and there might not be sufficient time to generate high enough levels of Gγ1-ΔCAAX to effectively inhibit the maternally derived product. If this explanation is correct, it would suggest that *gγ1* may also function at a later, *tre-1* independent step in the germ cell migration pathway since many of the germ cells in *nos-GAL4/UAS*-*Gγ1-ΔCAAX* embryos fail to coalesce with the SGPs.
(4.2 MB TIF)

## References

[1] Kunwar PS, Siekhaus DE, Lehmann R (2006). In vivo migration: a germ cell perspective.. Annu Rev Cell Dev Biol.

[2] Molyneaux K, Wylie C (2004). Primordial germ cell migration.. Int J Dev Biol.

[3] Boyle M, DiNardo S (1995). Specification, migration and assembly of the somatic cells of the Drosophila gonad.. Development.

[4] Boyle M, Bonini N, DiNardo S (1997). Expression and function of clift in the development of somatic gonadal precursors within the Drosophila mesoderm.. Development.

[5] Strome S, Lehmann R (2007). Germ versus soma decisions: lessons from flies and worms. Science..

[6] Mahowald AP (2001). Assembly of the Drosophila germ plasm.. Int Rev Cytol.

[7] Starz-Gaiano M, Cho NK, Forbes A, Lehmann R (2001). Spatially restricted activity of a Drosophila lipid phosphatase guides migrating germ cells.. Development.

[8] Zhang N, Zhang J, Purcell KJ, Cheng Y, Howard K (1997). The Drosophila protein Wunen repels migrating germ cells.. Nature.

[9] Van Doren M, Broihier HT, Moore LA, Lehmann R (1998). HMG-CoA reductase guides migrating primordial germ cells.. Nature.

[10] Deshpande G, Swanhart L, Chiang P, Schedl PD (2001). Hedgehog signaling in germ cell migration.. Cell.

[11] Chen Y, Struhl G (1998). In vivo evidence that Patched and Smoothened constitute distinct binding and transducing components of a Hedgehog receptor complex.. Development.

[12] Murone M, Rosenthal A, de Sauvage FJ (1999). Hedgehog signal transduction: from flies to vertebrates.. Exp Cell Res.

[13] Alcedo J, Zou Y, Noll M (2000). Posttranscriptional regulation of smoothened is part of a self-correcting mechanism in the Hedgehog signaling system.. Mol Cell.

[14] Deshpande G, Schedl P (2005). HMGCoA reductase potentiates hedgehog signaling in Drosophila melanogaster.. Dev Cell.

[15] Santos AC, Lehmann R (2004). Isoprenoids control germ cell migration downstream of HMGCoA reductase.. Dev Cell.

[16] Edwards PA, Ericsson J (1999). Sterols and isoprenoids: signaling molecules derived from the cholesterol biosynthetic pathway.. Annu Rev Biochem.

[17] Yi P, Han Z, Li X, Olson E (2006). The mevalonate pathway controls heart formation in Drosophila by isoprenylation of Gγ1.. Science.

[18] Olson EN (2006). Gene regulatory networks in the evolution and development of the heart.. Science.

[19] Jamora C, Yamanouye N, Van Lint J, Laudenslager JM, Vandenheede JR (1999). Gβγ-mediated regulation of Golgi organization is through the direct activation of protein kinase D.. Cell.

[20] Anel AMD, Malhotra V (2005). PKCn is required for β1γ2/β3γ2 and PKD-mediated transport to the cell surface and the organization of the Golgi apparatus.. J Cell Biol.

[21] Bard F, Malhotra V (2006). The formation of TGN to plasma membrane transport carriers.. Annu Rev Cell Dev Biol.

[22] Porter JA, Ekker SC, Park W-J, von Kessler DP, Young KE (1996). Hedgehog patterning activity: role of a lipophlic modification mediated by the carboxy-terminal autoprocessing domain.. Cell.

[23] Taylor AM, Nakano Y, Mohler J, Ingham PW (1993). Contrasting distributions of patched and hedgehog proteins in the Drosophila embryo.. Mech Dev.

[24] Tabata T, Kornberg TB (1994). Hedghog is a signaling protein with a key role in patterning Drosophila imaginal discs.. Cell.

[25] Burke R, Nellen D, Bellotto M, Hafen E, Senti KA (1999). Dispatched, a novel sterol-sensing domain protein dedicated to the release of cholesterol-modified hedghog from signaling cells.. Cell.

[26] Gallet A, Rodriguez R, Ruel R, Therond PP (2003). Cholesterol modification of Hedgehog is required for trafficking and movement, revealing and asymmetric cellular response to Hedgehog.. Dev Cell.

[27] Felsenfeld AL, Kennison JA (1995). Positional signaling by hedgehog in Drosophila imaginal disc development.. Development.

[28] Izumi Y, Ohta N, Itoh-Furuva A, Fuse N, Matsuzaki F (2004). Differential functions of G protein and Baz-aPKC signaling pathways in Drosophila neuroblast asymmetric division.. J Cell Biol.

[29] Denef N, Neubuser D, Perez L, Cohen SM (2000). Hedgehog induces opposite changes in turnover and subcellular localization of patched and smoothened.. Cell.

[30] Zhu AJ, Zheng L, Suyama K, Scott MP (2003). Altered localization of Drosophila Smoothened protein activates Hedgehog signal transduction.. Genes Dev.

[31] Michaelson D, Ahearn I, Bergo M, Young S, Philips M (2002). Membrane trafficking of heterotrimeric G proteins via the endoplasmic reticulum and Golgi.. Mol Biol Cell.

[32] Schillo S, Belusic G, Hartman K, Franz C, Kuhl B (2004). Targeted mutagenesis of the farnesylation site of Drosophila Gγe disrupts membrane association of the G protein βγ complex and affects the light sensitivity of the visual system.. J Biol Chem.

[33] Paulus H (2000). Protein splicing and related forms of protein autoprocessing.. Annu Rev Biochem.

[34] Mann RK, Beachy PA (2004). Novel lipid modifications of secreted protein signals.. Annu Rev Biochem.

[35] Lee JD, Treisman JE (2001). Sightless has homology to transmembrane acyltransferases and is required to generate active Hedgehog protein.. Curr Biol.

[36] Porter JA, Ekker SC, Park W-J, von Kessler DP, Young KE (1996). Hedgehog patterning activity: role of a lipophlic modification mediated by the carboxy-terminal autoprocessing domain.. Cell.

[37] Dawber RJ, Hebbes S, Herpers B, Docquier F, van den Heuvel M (2005). Differential range and activity of various forms of the Hedgehog protein.. BMC Dev Biol.

[38] Gallet A, Ruel L, Staccini-Lavenant L, Therond PP (2005). Cholesterol modification is necessary for controlled planar long-range activity of Hedgehog in *Drosophila* epithelia.. Development.

[39] Callejo A, Torroja C, Quijada L, Guerrero I (2007). Hedgehog lipid modifications are required for Hedgehog stablization in the extracellular matrix.. Development.

[40] Caspary T, Garcia-Garcia MJ, Huangfu D, Eggenschwiller JT, Wyler MR (2002). Mouse dispatched homolog1 is required for long-range, but not juxtacrine, Hh signaling.. Curr Biol.

[41] Gilse B, Miller CA, Crozatier M, Halbise MA, Wise S (2005). Shifted, the Drosophila ortholog of Wnt inhibitory factor-1, controls the distribution and movement of Hedgehog.. Dev Cell.

[42] Gorfinkiel N, Sierra J, Callejo A, Ibanez C, Guerrero I (2005). The Drosophila ortholog of the human Wnt inhibitor factor Shifted controls the diffusion of lipid-modified Hedgehog.. Dev Cell.

[43] Perrimon N, Hacker U (2004). Wingless, hedgehog and heparan sulfate proteoglycans.. Development.

[44] Han C, Belenkaya TY, Khodoun M, Tauchi M, Lin X (2004). Distinct and collaborative roles of Drosophila EXT family proteins in morphogen signalling and gradient formation.. Development.

[45] The I, Bellaiche Y, Perrimon N (1999). Hedgehog movement is regulated through tout velu-dependent synthesis of a heparan sulfate proteoglycan.. Mol Cell.

[46] Franch-Marro X, Marchand O, Piddini E, Ricardo S, Alexandre C (2005). Glypicans shunt the wingless signal between local signaling and further transport.. Development.

[47] Marois E, Mahmoud A, Eaton S (2006). The endocytic pathway and formation of the wingless morphogen gradient.. Development.

[48] Chen CA, Manning DR (2001). Regulation of G proteins by covalent modification.. Oncogene.

[49] Simmonds AJ, dosSantos G, Livne-Bar I, Karuse HM (2001). Apical localization of *wingless* transcripts is required for *wingless* signaling.. Cell.

[50] Le-Niculesu H, Niesman I, Fisher T, DeVries L, Farquhar MG (2005). Identification and characterization of GIV, a novel Gα_i/s_-interacting protein found on COPI, endoplasmic reticulum that concentrates proteins involved in COPII vesicle biogenesis.. J Biol Chem.

[51] Deshpande G, Stukey J, Schedl P (1995). *scute* (*sis-b*) function in *Drosophila* sex determination.. Mol Cell Biol.

[52] Kunwar PS, Sano H, Renault AD, Barbosa V, Fuse N, Lehmann R (2008). Tre1 GPCR initiates germ cell transepithelial migration by regulating Drosophila melanogaster E-cadherin.. J Cell Biol.

