# Supplementary figures and images for: Gγ1, a Downstream Target for the hmgcr-Isoprenoid Biosynthetic Pathway, Is Required for Releasing the Hedgehog Ligand and Directing Germ Cell Migration
Source: PLoS Genet. 2009 Jan 9;5(1):e1000333. doi: 10.1371/journal.pgen.1000333 (PMC2607556; doi:10.1371/journal.pgen.1000333)

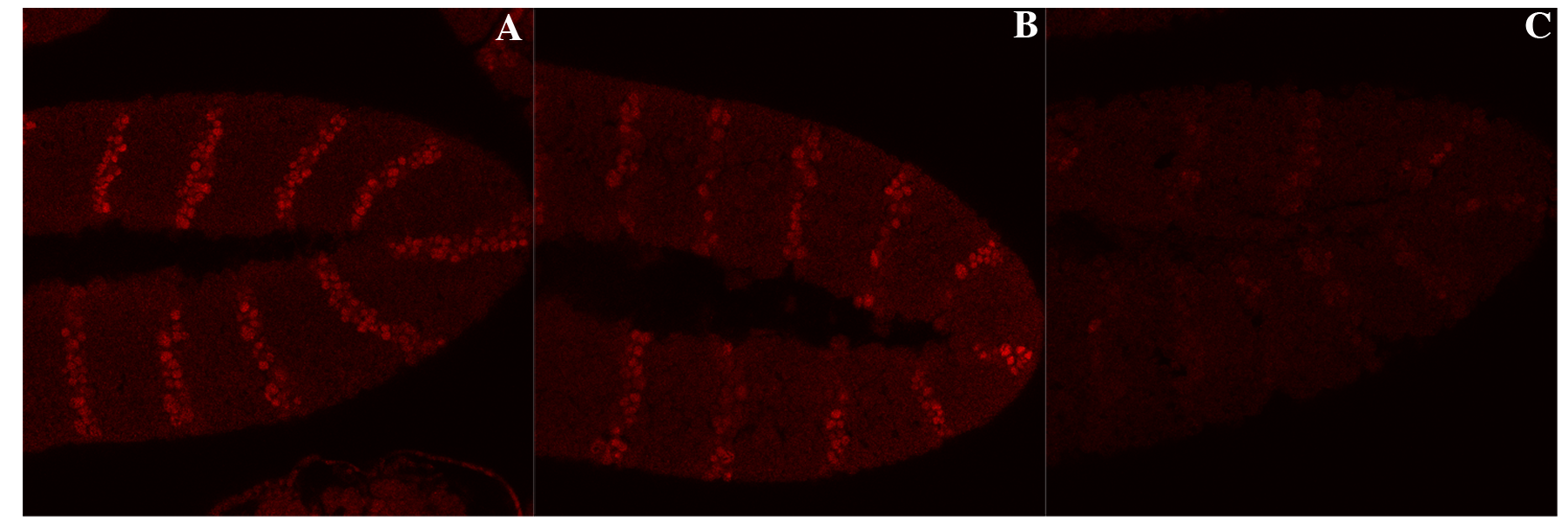

Supplement: Figure S1 — Engrailed expression is not properly maintained in gγ1 mutant embryos. Embryos from the gγ1N159/ Cy0, en:LacZ stock were collected and fixed using standard procedure. Embryos were genotyped by simultaneously staining them with β-galactosidase (imaged in green: not shown) and En (imaged in red) antibodies. Balancer embryos (Panel A) show strong En specific expression in 14 stripes. By contrast, En specific signal starts to decline by Stage 11 in the homozygous gγ1N159 embryos (Panels B and C). As illustrated in these two panels there is some variation in the extent of the reduction in En expression. In some embryos, moderate levels of En protein are detected (B) while in others only low levels are observed (C). Because of the variability in En accumulation in gγ1N159 homozygous embryos, we classified the En staining pattern. For the heterozygous gγ1N159/+ control, 6/7 embryos had high levels of En accumulation, while 1 embryo had a medium level of accumulation. For the homozygous gγ1N159 embryos 4/11 (37%) had little En protein (like the example shown in the figure) while 4/11 had a medium level of En protein (like the example shown in the figure). The 3 remaining embryos (27%) resembled wild type. We also examined En expression in homozygous gγ1 k0817 embryos. In this experiment all of the heterozygous gγ1 k0817/+ control embryos had a high level of En protein (9 embryos). For the homozygous gγ1 k0817 mutant embryos 8/18 (44%) had a low level of En protein, while 5/18 (28%) had a medium level of En protein. Finally, 5/18 (28%) homozygous mutant embryos had a high level of En protein. (3.2 MB TIF) [file pgen.1000333.s001.tif]

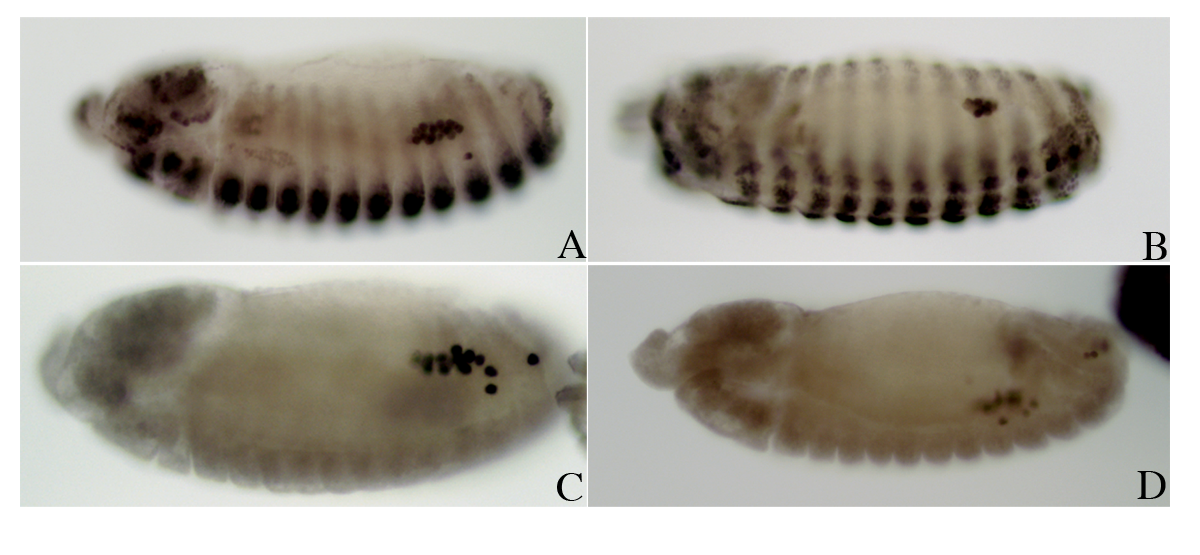

Supplement: Figure S2 — Germ cell migration defects are also observed in gγ1 k0817 mutant embryos. Embryos from a gγ1 k0817/Cy0 en:LacZ stock were probed with Vasa to mark the germ cells and β-galactosidase antibodies to identify heterozygous and homozygous mutant embryos. Panels A and B are gγ1 k0817/Cy0 en:LacZ embryos (note β-galactosidase expression) while panel C and D are mutant. Panel C shows embryo with 3 scattered cells whereas the embryo in panel D has more than 6 scattered germ cell cells. About 15% (4/22) of the mutant embryos had 3–4 scattered germ cells (example in panel C), while about 40% (8/22) of the mutant embryos had 5 or more scattered germ cells (example in panel D). The remaining embryos (10/22 or 45%) had 2 or fewer scattered germ cells. (2.0 MB TIF) [file pgen.1000333.s002.tif]

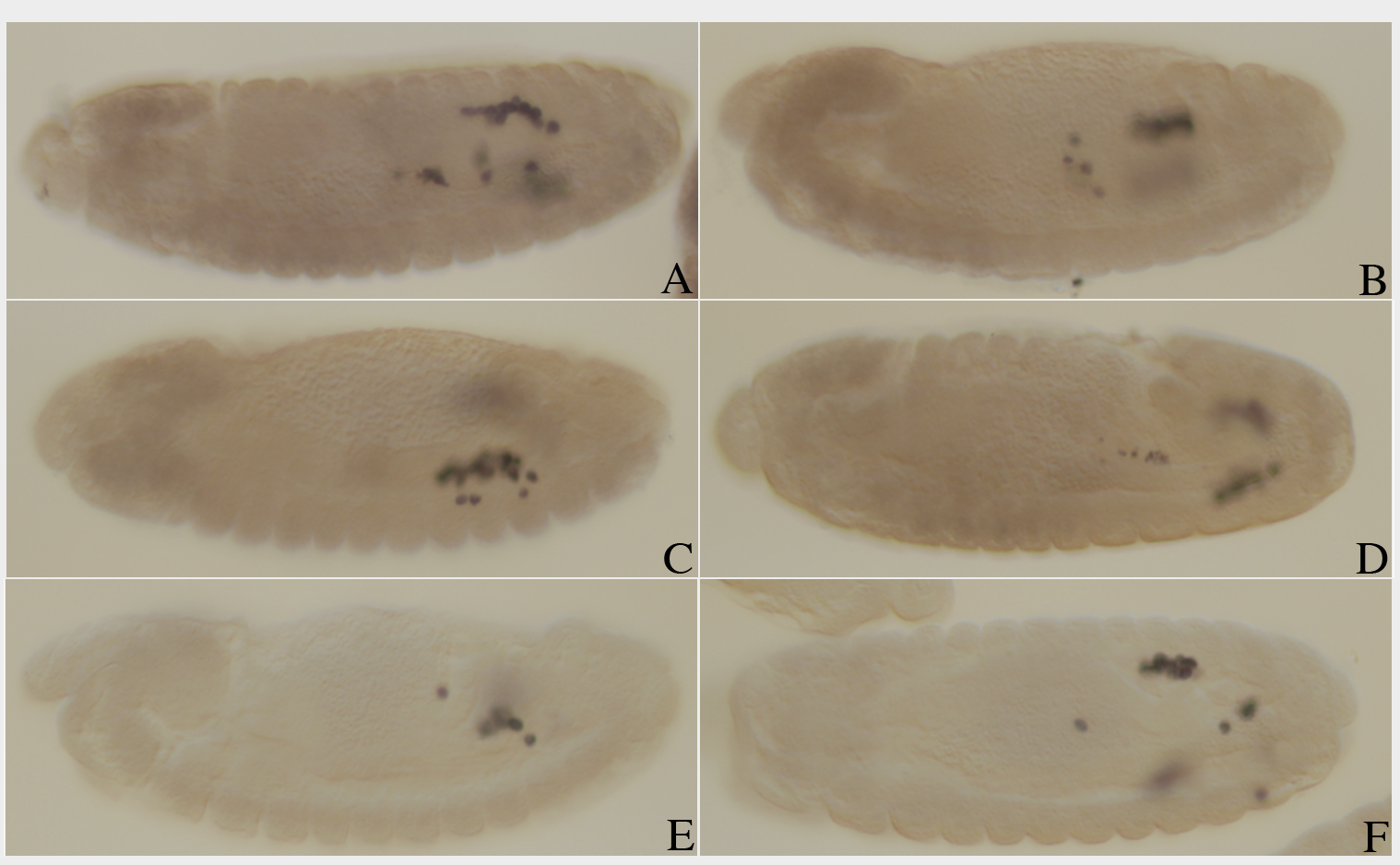

Supplement: Figure S3 — Ectopic expression of gγ1-ΔCAAX in the mesoderm and in germ cells disrupts germ cell migration. Panels A–F show stage 13–15 twist-GAL4/UAS-Gγ1-ΔCAAX or nos-GAL4/UAS-Gγ1-ΔCAAX embryos probed with Vasa antibodies to visualize migrating germ cells. Panels A–D: Germ cell migration defects in twist-GAL4/UAS-Gγ1-ΔCAAX embryos. Panels E and F: Germ cell migration defects in nos-GAL4/UAS-Gγ1-ΔCAAX embryos. Recent studies by Kunwar et al. [52] on germ cell migration have suggested that gγ1 has a cell autonomous requirement in germ cells. To test the cell autonomous function of gγ1 in germ cell migration, these authors rescued the gastrulation defects of progeny from gγ1 germline clone mothers using a nullo-GAL4 to drive expression of a UAS-gγ1 transgene. They reported that the pole cells in these embryos failed to migrate properly out of the midgut and exhibited phenotypes similar to those found for mutations in the G protein-coupled receptor (GPCR) gene trapped in endoderm 1 (tre1). We wondered whether the geranylated form of Gγ1 is also required in the germline. To explore this possibility we ectopically expressed the dominant negative gγ1 deletion mutant Gγ1-ΔCAAX in the germline. As shown in Figure S3E and S3F, ectopic expression of the Gγ1-ΔCAAX protein in germ cells using a nos-GAL4 driver disrupts germ cell migration. We found that nearly 60% of the stage 13–15 nos-GAL4/UAS-Gγ1-ΔCAAX embryos had 3 or more lost germ cells, while 33% had 5 or more lost germ cells (n = 100 embryos). This is roughly equivalent to the germ cell migration defects evident when Gγ1-ΔCAAX is expressed in the mesoderm using a twist-GAL4 driver. The effects of the dominant negative protein in germ cells would support the findings of Kunwar et al., and argue that gγ1 (specifically geranylated Gγ1) has a cell autonomous function in these cells during their migration towards the SGPs. On the other hand, our results differ somewhat from those reported by Kunwar et al. in that we did not observe any [file pgen.1000333.s003.tif]
